# Metformin and Resveratrol Inhibited High Glucose-Induced Metabolic Memory of Endothelial Senescence through SIRT1/p300/p53/p21 Pathway

**DOI:** 10.1371/journal.pone.0143814

**Published:** 2015-12-02

**Authors:** Erli Zhang, Qianyun Guo, Haiyang Gao, Ruixia Xu, Siyong Teng, Yongjian Wu

**Affiliations:** State Key Laboratory of Cardiovascular Disease, Department of Cardiology, Cardiovascular Institute, Fuwai Hospital, and National Center for Cardiovascular Diseases, Chinese Academy of Medical Sciences and Peking Union Medical College, Beijing, China; Niigata University Graduate School of Medical and Dental Sciences, JAPAN

## Abstract

Endothelial senescence plays crucial roles in diabetic vascular complication. Recent evidence indicated that transient hyperglycaemia could potentiate persistent diabetic vascular complications, a phenomenon known as “metabolic memory.” Although SIRT1 has been demonstrated to mediate high glucose-induced endothelial senescence, whether and how “metabolic memory” would affect endothelial senescence through SIRT1 signaling remains largely unknown. In this study, we investigated the involvement of SIRT1 axis as well as the protective effects of resveratrol (RSV) and metformin (MET), two potent SIRT1 activators, during the occurrence of “metabolic memory” of cellular senescence (senescent “memory”). Human umbilical vascular endothelial cells (HUVECs) were cultured in either normal glucose (NG)/high glucose (HG) media for 6 days, or 3 days of HG followed by 3 days of NG (HN), with or without RSV or MET treatment. It was shown that HN incubation triggered persistent downregulation of deacetylase SIRT1 and upregulation of acetyltransferase p300, leading to sustained hyperacetylation (at K382) and activation of p53, and subsequent p53/p21-mediated senescent “memory.” In contrast, senescent “memory” was abrogated by overexpression of SIRT1 or knockdown of p300. Interestingly, we found that SIRT1 and p300 could regulate each other in response to HN stimulation, suggesting that a delicate balance between acetyltransferases and deacetylases may be particularly important for sustained acetylation and activation of non-histone proteins (such as p53), and eventually the occurrence of “metabolic memory.” Furthermore, we found that RSV or MET treatment prevented senescent “memory” by modulating SIRT1/p300/p53/p21 pathway. Notably, early and continuous treatment of MET, but not RSV, was particularly important for preventing senescent “memory.” In conclusion, short-term high glucose stimulation could induce sustained endothelial senescence via SIRT1/p300/p53/p21 pathway. RVS or MET treatment could enhance SIRT1-mediated signaling and thus protect against senescent “memory” independent of their glucose lowering mechanisms. Therefore, they may serve as promising therapeutic drugs against the development of “metabolic memory.”

## Introduction

The prevalence of diabetes has been steadily rising in recent decades. In 2010, an estimated 285 million people worldwide had diabetes mellitus, and this number is projected to rise to 439 million by 2030 [1]. A large body of evidence indicates that long-term hyperglycaemia in diabetic patients may induce a variety of disabling and life-threatening vascular complications, including microvascular complications (e.g. diabetic nephropathy and retinopathy) and macrovascular complications (e.g. cardiovascular diseases), resulting in amputation, blindness, myocardial infarction, stroke, and hypertension. Owing to its high prevalence, diabetic vascular complications have become a global health burden.

One of the major contributors in the development of diabetes-associated cardiovascular diseases is endothelial senescence, a permanent arrest of cellular growth and proliferation. A number of *in vitro* and in *vivo studies* have shown that atherosclerosis is an age-related chronic disease, in which cellular aging or premature senescence of endothelium plays key roles in promoting vascular inflammation and dysfunction [2]. High blood glucose in diabetes patients has been demonstrated to be a crucial factor accelerating the progression of endothelial senescence. Substantial evidence indicated that hyperglycaemia-induced production of reactive oxygen species (ROS) may promote telomere shortening and DNA damage, trigger a p53-dependent damage response, impair the repair capacity of endothelial lining, lead to a more pro-inflammatory, pro-atherosclerotic, and pro-thrombotic phenotype, and thus accelerate the development of diabetic vascular complications [2–5].

As is well known, p53 is a crucial transcription activator, and it induces the cyclin-dependent inhibitor p21, an important negative regulator of cell proliferation, to promote cellular senescence [6]. Recent evidence shows that the p53 protein is controlled by many different forms of post-translational modifications, including ubiquitylation, phosphorylation, acetylation, sumoylation, methylation, and neddylation. Acetylation of p53, in particular, has profound effects on its transcriptional activity, because it increases p53 protein stability, binding to low affinity promoters, and association with other transcription factors. In terms of cellular senescence, acetylation of p53 at K120, K320, and K382 has been suggested to be essential for the transcriptional activation of p21 [7].

Generally, the delicate balance of p53 acetylation status is regulated by two groups of enzymes, histone acetyltransferases (HATs) and histone deacetylases (HDACs). P300 is among the most known HATs associated with p53 acetylation. Studies have suggested that p300 can directly bind to p53 and acetylate the same lysine residues. Of importance, p300-mediated acetylation of p53 at K320 and K382 in human cancer cell lines not only increased the transcriptional activity of p53, but also promoted the recruitment of its co-activators to the p21 promoter, thus enhancing p21 transcription following DNA damage [8–10]. On the contrary, deacetylation of p53 by HDACs has been demonstrated to down-regulate its transcriptional activity and promote cell growth, proliferation, and survival. Sirtuin 1 (SIRT1), the closest mammalian homologue of Sir2, is an important negative regulator of p53-mediated pathways. It was shown that SIRT1 acted specifically on K382, and that deacetylation of p53 at K382 by SIRT1 inhibited p21 activation and subsequent cellular senescence in response to DNA damage [11–13]. In the context of diabetic vascular complications, decreased expression of SIRT1 and increased acetylation of p53 at SIRT1-specific K382 has been found to mediate high glucose-induced endothelial senescence [14–17]. Therefore, p300 and SIRT1 may be essential for high glucose-induced cellular senescence by modulating the acetylation status and transactivation activity of p53.

Recently, a memory phenomenon of high glucose has been observed in both experimental models and clinical studies, such as the Diabetes Control and Complications Trial (DCCT) and Epidemiology of Diabetes Interventions and Complications (EDIC) study in type I diabetes (T1D) patients and The UK Prospective Diabetes Study (UKPDS) in T2D patients. It was indicated that prior exposure of target cells to high glucose may induce persistent harmful effects even long after attainment of glycaemic control. This phenomenon was termed as “metabolic memory”. HATs and HDACs were shown to mediate “metabolic memory” through epigenetic mechanisms, such as acetylation and deacetylation of particular histone proteins [18]. However, so far to our knowledge, there is no evidence that acetylation of non-histone proteins, such as p53, may convey the same “memory” phenomenon on cellular senescence. Hence, it would be interesting to know whether SIRT1- and/or p300-modulated acetylation of p53 was involved in the occurrence of “metabolic memory” of endothelial senescence (senescent “memory”).

Metformin (MET) and Resveratrol (RSV) are two potent activators of SIRT1. While metformin is the most widely prescribed oral hypoglycaemic drug for the treatment of T2D, resveratrol is a natural, biologically active polyphenol widely present in dietary sources and supplements. Previously, mounting evidence suggested that both of MET and RSV could attenuate hyperglycaemia-induced endothelial senescence in cellular and animal models, and confer strong protection against diabetes and diabetic vascular complications in clinical settings [17,19–24]. However, whether MET and RSV may exert preventive and/or therapeutic effects on “metabolic memory” remains largely unknown. Therefore, in this study, we also tested the hypothesis that metformin and resveratrol may modulate high glucose-induced “metabolic memory” of cellular senescence in human umbilical vein endothelial cells (HUVECs) via SIRT1-mediated signaling pathways.

## Methods

### Endothelial cell culture and drug treatment protocols

Human umbilical vein endothelial cells (HUVECs) (ScienCell, CA, USA) were cultured in endothelial cell medium (ECM) (ScienCell, CA, USA), supplemented with 5% fetal bovine serum (FBS), 1% endothelial cell growth supplement (ECGS) (ScienCell, CA, USA), 100 U/mL penicillin, and 100 U/mL streptomycin. Cells are cultured at 37°C with 5% CO_2_. The cellular model of senescent “memory” was built according to a modified method described before [25]. Briefly, at passage 5 to 7, HUVECs were subcultured in 5 mM normal D-glucose for 6 days (NG group), 30 mM high glucose for 6 days (HG group), or 3 days of HG followed by another 3 days of NG (HN group) in the presence or absence of resveratrol (RSV; Sigma-Aldrich, St. Louis, MO, USA) and metformin (MET; Sigma-Aldrich, St. Louis, MO, USA). For studies determining the effects of osmotic pressure on metabolic memory, 25 mM D-mannitol (Sigma-Aldrich, St. Louis, MO, USA) was added into 5 mM normal glucose medium to reach a final 30 mM. Unless specified, for studies determining the drug effects on preventing senescent “memory”, HN-treated HUVECs were supplemented with RSV or MET during the entire period of sequential HG and NG incubation. For the studies determining the effects of drug start time and duration on senescent “memory”, HN-treated HUVECs were supplemented with RSV or MET during the first 3 days in HG incubation (RSV_1-3_ or MET_1-3_), the last 3 days in NG incubation (RSV_4-6_ or MET_4-6_), or the whole 6 days in sequential HG and NG incubation (RSV_1-6_ or MET_1-6_). Regardless the type of media (NG or HG) or drug supplementation (MET or RSV) mentioned above, media was always changed every 24 hours.

### Plasmid and small interfering RNA (siRNA) transfection

A recombinant plasmid overexpressing SIRT1 cDNA (pSIRT1; NM_012238.4) was purchased from GenePharma (Shanghai, China). Commercially available human SIRT1 siRNA (si-SIRT1), p300 siRNA (si-p300), AMPK siRNA (si-AMPK) and non-targeted scrambled siRNAs were obtained from Santa Cruz (Dallas, USA). Briefly, HUVECs were transiently transfected with plasmid or siRNA using Opti-MEM I reduced serum media and Lipofectamine 3000 (Invitrogen, USA) or Lipofectamin RNAiMAX (Invitrogen, USA) according to manufacturer’s instructions. For SIRT1 overexpression and p300 knockdown assay, pSIRT1 or si-p300 was transfected into HN-treated cells for two times, 12 hours before HG and NG incubation, respectively. For SIRT1 and AMPK knockdown assay, si-SIRT1 or si-AMPK was transfected into RSV- or MET-treated cells twice, 12 hours before HG and NG incubation, respectively. For assays testing the protective effects of anti-diabetic drugs against senescent “memory”, RSV or MET was supplemented 12 hours after transfection.

### Western blotting

Briefly, 20 μg of cellular proteins were subjected to SDS-PAGE (NuPAGE precast gel, Thermo Fisher Scientific, USA) for electrophoresis and followed by dry transfer onto nitrocellulose membranes (iBlot Transfer Stack, Thermo Fisher Scientific, USA). The membranes were blocked with 5% BSA in TBS containing 0.1% (v/v) Tween 20 (Sigma-Aldrich, St. Louis, MO, USA) for 60 minutes and incubated with the following primary antibodies: anti-human SIRT1 (1:2000 dilution), acetylated-p53 at K382 (Ac-p53; 1:500 dilution), AMPKα (1:4000 dilutuion), and p21 (1:2000 dilution) antibodies purchased from Cell Signaling Technology (Beverly, MA, USA), and p300 (1:2000 dilution) and total p53 (1:2000 dilution) from Santa Cruz (Dallas, USA). Anti-human GAPDH (1:5000 dilution), and β-actin (1:10000 dilution) antibodies were used as loading controls and purchased from Millipore (Billerica, USA) and Sigma Aldrich (St Louis, USA), respectively. Anti-rabbit and anti-mouse secondary antibodies (1:10000 dilutions) were bought from GE Healthcare (Buckinghamshire, United Kingdom). The immunoreactive bands were detected by an enhanced chemiluminescence system (Proteinsimple, Santa Clara, USA). Related signals were quantified using Proteinsimple image software (Santa Clara, USA).

### SIRT1 deacetylase activity

SIRT1 deacetylase activity was quantified using the fluorometric SIRT1 Assay kit (Sigma-Aldrich, St. Louis, USA) according to the manufacturer's instructions. Fluorescence intensity at 444 nm (excitation 355 nm) was recorded and normalized to micrograms of protein. Values are represented as -fold of NG control.

### Senescence-associated β-galactosidase staining

The senescence-associated β-galactosidase (SA β-Gal) activity was determined in formaldehyde-fixed cells according to the manufacturer’s instructions (Sigma-Aldrich, St. Louis, USA). The assay is based on a histochemical stain for β-galactosidase activity at pH 6 and found to be specific for senescent endothelial cells, but not for quiescent or terminally differentiated cells. Briefly, cells were then washed once with PBS after media removal, and then fixed with the fixation buffer for 7 min at room temperature. After washing twice with PBS, the staining solution (pH 6) was added to each well. Cells were incubated at 37°C overnight and then observed and photographed under a microscope (DM IL LED, Leica). The amount of blue cells (positive cells) and total cells in each field of view were counted using Image J software (NIH).

### Measurement of intracellular reactive oxygen species (ROS)

The level of intracellular ROS was quantified by using Reactive Oxygen Species Assay Kit according to the manufacturer’s instructions (Beyotime, China). Briefly, after HG, NG and/or drug treatment, HUVECs were washed with serum-free media for three times, incubated with 10 μM of DCFH-DA, and kept in dark for 20 min at 37 degree. Cells were then washed twice with cold PBS. The qualitative analysis of ROS generation was done using a fluorescence microscope with excitation at 488 nm and emission at 525 nm.

### Statistical analysis

Results were presented as mean ± SEM. All data were analysed by statistical software ‘GraphPad Prism 5.0’ (San Diego, CA, USA). Statistical analysis was performed using one-way ANOVA. *Post hoc* comparisons between the groups were performed by Bonferroni multiple comparisons test. In Figures, significance is expressed by *P* < 0.05. All experiments were repeated three or more times.

## Results

### Transient high glucose induced persistent cellular senescence through SIRT1/p300/p53/p21 pathway

**Fig 1:**
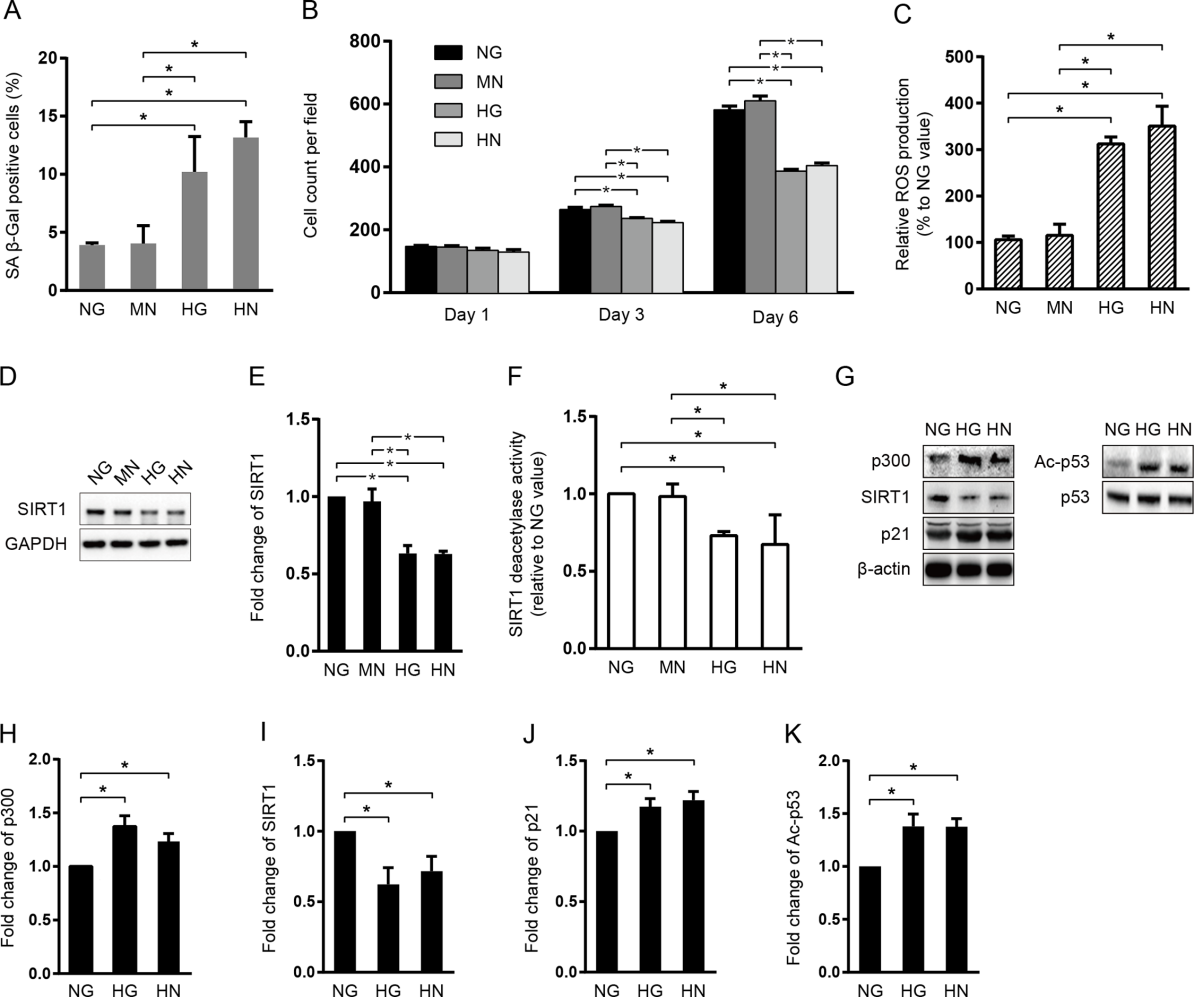
Effect of the HG-induced “memory” on cell growth and aging in HUVECs. HUVECs were cultured in normal glucose media (D-glucose, 5 mM) for 6 days (NG), high glucose media (30 mM) for 6 days (HG), 3 days of normal glucose supplemented with D-mannitol (25 mM) followed by another 3 days of normal glucose media (MN), or 3 days of high glucose followed by another 3 days of normal glucose media (HN). (A) Cells were fixed and stained for SA β-Gal activity. Histogram represent the percentage of SA β-Gal positive cells (n = 5 image per group). (B) Cell count per microscopic field at different time points during cell subculture. (n = 3 image per group) (C) Relative ROS production. (D-E) Immunoblotting and quantification of SIRT1 expression. Values were normalized to GAPDH. (F) SIRT1 deacetylase activtity. (G-K) Immunoblotting and quantification of p300, SIRT1, p21, and Ac-p53 protein expression. For p300, SIRT1, and p21, values were normalized to β-actin; for Ac-p53, normalized to total p53. Value was normalized to total p53. *P < 0.05.

To explore whether transient high glucose could induce persistent endothelial senescence, a cellular model of senescent “memory”, HUVECs were subcultured in three different types of endothelial cell (EC) media: normal glucose (NG; 5 mM, 6 days), high glucose (HG; 30 mM, 6 days), or high glucose followed by normal glucose media (HN; 3 days of HG + 3 days of NG). Cellular senescence in HUVECs was then determined by senescence associated β-Galactosidase (SA β-Gal) assay (Fig 1A). Compared with NG, both HG and HN significantly increased the percentage of SA β-Gal staining cells by more than 2 fold (*P < 0*.*05*; Fig 1A). Although HN group exhibited higher average percentage of SA β-Gal positivity, no statistical difference was observed between HG- and HN-treated HUVECs. To rule out the possibility that the increased SA β-Gal staining obsertved in HN group was due to higher osmotic pressure during HG subculture, we incubated HUVECs in 5 mM NG media supplemented with 25 mM D-mannitol for 3 days, and then changed to NG media for another 3 days (MN). It was shown that incubation of HUVECs in MN didn’t increase SA β-Gal staining compared with NG group. In contrast, significantly higher SA β-Gal positive cells were presented in HG- or HN-treated HUVECs, compared with MN-treated cells (*P < 0*.*05*; Fig 1A). In addition to SAβ-Gal staining, we also counted cell number under microscope at 1^st^, 3^rd^, and 6^th^ day of cell subculture to further confirm cell growth arrest. We found that the cell counts at day 3 and day 6 were significantly lower in HN treated group compared to either NG or MN treated group (*P < 0*.*05*; Fig 1B). These results indicated that prior HG stimulation was strong enough to induce enhanced endothelial senescence and reduced cell growth in HUVECs despite subsequent restoration to NG for the same period of time.

Since oxidative stress has been suggested in many models of HG-mediated cellular aging, we investigated the possible alteration in reactive oxygen species (ROS) generation. It was not surprised that 6 days of HG treatment increased intracellular ROS production in HUVECs. Interestingly, HN treatment also provoked significantly higher level of ROS in comparison to NG or MN (Fig 1C), suggesting that ROS generation was associated with accelerated cellular aging.

Subsequently, the effect of HN treatment on SIRT1 and p300, as well as their downstream signaling targets was investigated (Fig 1D–1K). Compare to either NG or MN treated cells, exposure to HG resulted in a significant decrease in SIRT1 protein expression (*P < 0*.*05*; Fig 1D and 1E) and its deacetylase activity (*P < 0*.*05*; Fig 1F), suggesting that prolonged alterations in SIRT1 function were caused by prior HG stimulation, and was not dependent on changes in osmotic pressure The protein expression of p300, acetylated p53 at K382 (Ac-p53), and p21 in HN group were significantly increased in comparison to their counterpart in NG group, and maintained at comparable levels to those in HG group (*P < 0*.*05*; Fig 1G–1K). These results revealed that sustained down-regulation of SIRT1 expression and deacetylase activity, as well as up-regulation of p300, Ac-p53, and p21 expression may be associated with senescent “memory” in HUVECs.

**Fig 2:**
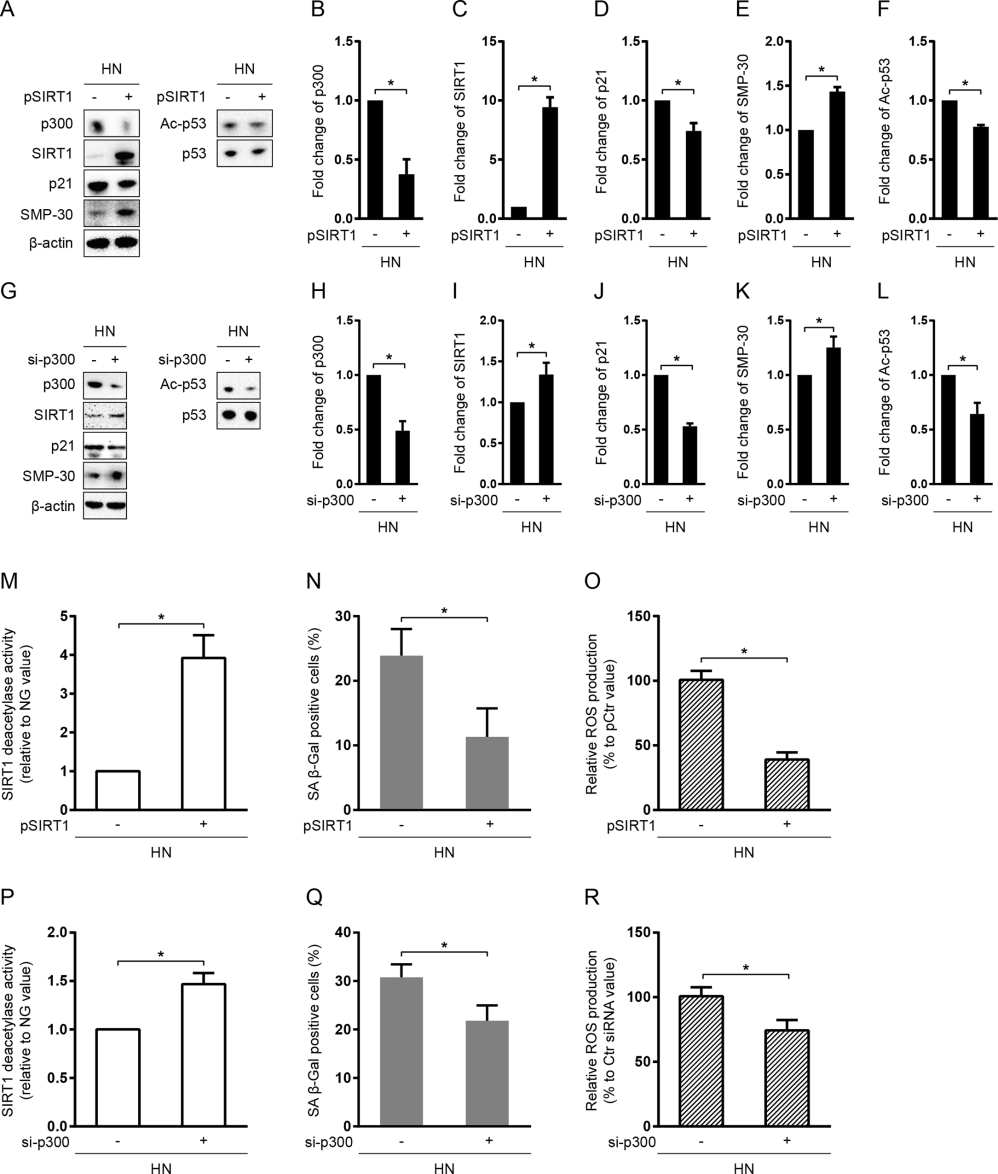
Overexpression of SIRT1 or suppression of p300 inhibited transient HG-induced senescent “memory” in HUVECs. Cells were cultured in HG for 3 days followed by 3 days of NG media. SIRT1 plasmid (pSIRT1) or p300 siRNA (si-p300) was transfected into HUVECs twice, 12 hours prior to HG and NG incubation, respectively. After 6 days of culture, cells were harvested for further analysis. Immunoblotting and quantification of p300, SIRT1, p21, Ac-p53, and p53 protein expression after pSIRT1 transfection (A-F) or after 300 siRNA transfection (G-L). For p300, SIRT1, and p21, values were normalized to β-actin; for Ac-p53, normalized to total p53. SIRT1 activity, SA β-Gal positivity, and relative ROS production in HUVECs transfected with pSIRT1 (M-O) or p300 siRNA (P-R). *P < 0.05.

To further determine whether SIRT1 and p300 regulate p53/p21 signaling and persistent senescent phenotype through modulation of p53 acetylation at K382, we transfected SIRT1 plasmid (pSIRT1) and p300 siRNA (si-p300) into HN-treated HUVECs, respectively. After transfection of pSIRT1, the protein expression and deacetylase activity of SIRT1 was significantly increased, while the protein expression of Ac-p53 and p21 were markedly decreased (*P < 0*.*05*; S1 Fig and Fig 2A–2F and 2M). In a similar manner, p300 siRNA transfection reduced the protein levels of p300, Ac-p53, and p21 (*P < 0*.*05*; S1 Fig and Fig 2G–2L). It is interesting that SIRT1 over-expression suppressed the expression of p300 by nearly 50% (*P < 0*.*05*; Fig 2A and 2B), and p300 knock-down reciprocally increased the expression and activity of SIRT1 (*P < 0*.*05*; Fig 2G, 2I and 2P).

Furthermore, the effect of SIRT1 overexpression and/or p300 knockdown on HUVEC senescence was determined by both anti-senescence marker protein-30 (SMP-30), a molecular marker, and SA β-Gal staining, a phenotypic marker of cellular senescence. It was shown that pSIRT1 transfection significantly increased the protein level of SMP-30 (*P < 0*.*05*; Fig 2A and 2E) and reduced the SA β-Gal staining in HN-treated HUVECs (*P < 0*.*05*; Fig 2N). Transfection of p300 siRNA also significantly suppressed HUVEC senescence (*P < 0*.*05*; Fig 2G, 2K and 2Q. Interestingly, SIRT1 overexpression and p300 knock down prevented HN-mediated ROS production, indicating that SIRT1 and p300 may be involved in feed-back regulations of HG-induced oxidative stress. Hence, these data collectively indicated that SIRT1/p300/p53/p21 pathway was the underling mechanism for the occurrence of senescent “memory” in HUVECs.

### Metformin and resveratrol reversed the “metabolic memory” of endothelial senescence through SIRT1-mediated signaling

**Fig 3:**
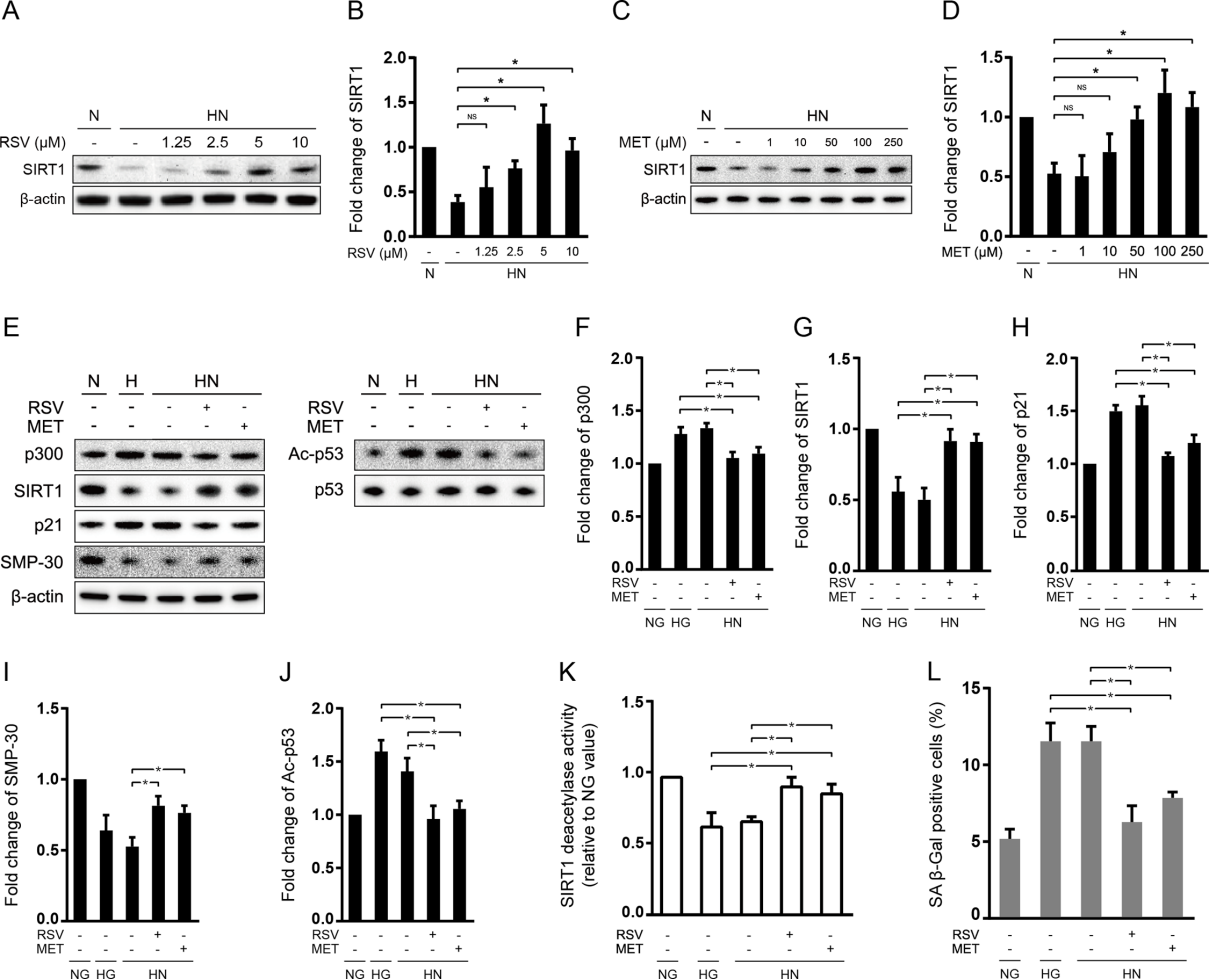
Effect of resveratrol and/or metformin on SIRT1-mediated signaling and senescent “memory” in HUVECs. Immunoblotting and quantification of SIRT1 protein expression after (A-B) resveratrol treatment (RSV; 0, 1.25, 2.5, 5, or 10 μM) or (C-D) metformin treatment (MET; 0, 1, 10, 50, 100, 250 μM). Values were normalized to β-actin. (E-J) Immunoblotting and quantification of p300, SIRT1, p21, Ac-p53, and p53 protein expression after RSV (5 μM) or MET (50 μM) treatment. For p300, SIRT1, and p21, values were normalized to β-actin; for Ac-p53, normalized to total p53. (K) The deacetylase activity of SIRT1 in HUVECs treated with or without RSV (5 μM) or MET (50 μM). (L) The percentage of SA β-Gal positive cells in HUVECs treated with or without RSV (5 μM) or MET (50 μM). *P < 0.05.

To explore whether potent SIRT1 activators regulate transient HG-induced senescent “memory” in ECs, HN-treated HUVECs were supplemented with resveratrol (RSV) or metformin (MET) during the entire 6-day cell subculture. First of all, we treated HUVECs measured the concentration-dependent expression of SIRT1 to determine the optimum dosage of RSV. We chose dosage ranges based on clinically achievable plasma concentration of RSV [26,27] and MET [28–30]. It was shown that, although SIRT1 protein expression started to increase at 2.5 μM RSV, the highest increase of SIRT1 expression was recorded at 5 μM (*P < 0*.*05*; Fig 3A and 3B). In a similar manner, 50 μM or higher MET treatment markedly increased SIRT1 expression (*P < 0*.*05*; Fig 3C and 3D). Accordingly, we used 5 μM RSV and 50 μM MET in subsequent experiments. When cells were treated with RSV or MET during the incubation in HG for 3 days followed by NG for another 3 days, the expression and deacetylase activity of SIRT1 were significantly increased and the expression of p300, Ac-p53, and p21 was significantly decreased (*P < 0*.*05*; Fig 3E–3K). In accordance to these changes, RSV or MET treatment also significantly increased the expression of SMP-30 (*P < 0*.*05*; Fig 3E and 3I) and decreased the percentage of SA β-Gal staining (*P < 0*.*05*; Fig 3L) in HN-treated HUVECs.

**Fig 4:**
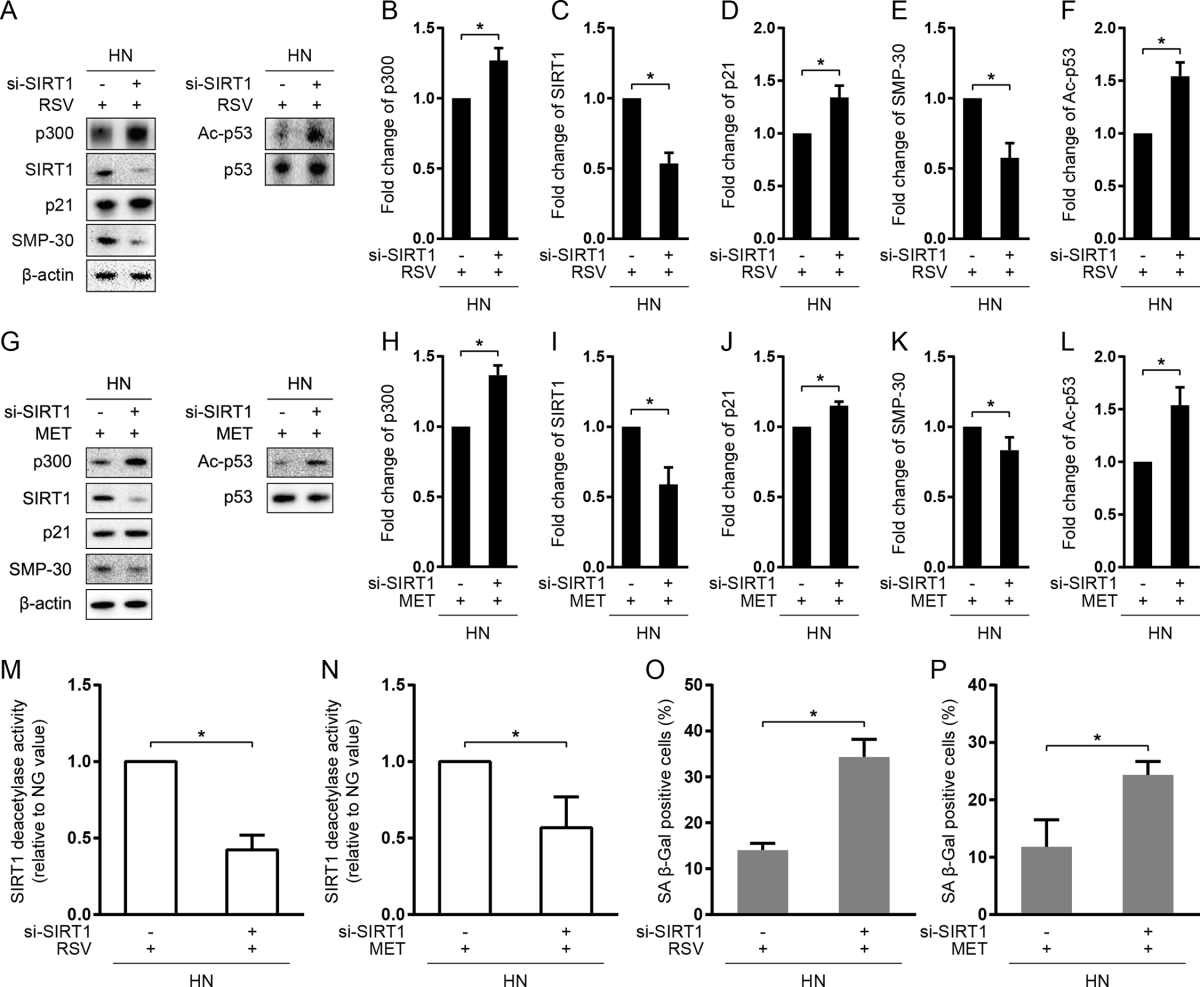
SIRT1 knockdown abolished the protective effects of resveratrol and metformin against the occurrence of senescent “memory”. HUVECs were transfected with SIRT1 siRNA (si-SIRT1) and incubated in HG for 3days and then NG for 3 days. Resveratrol (RSV) or metformin (MET) was added into culture media during the entire 6 days of subculture. Immunoblotting and quantification of p300, SIRT1, p21, Ac-p53, and p53 protein expression with RSV (A-F) or MET treatment (G-L). For p300, SIRT1, and p21, values were normalized to β-actin; for Ac-p53, normalized to total p53. The deacetylase activity of SIRT1 with RSV (M) or MET (N). The percentage of SA β-Gal positive cells with RSV (O) or MET (P). *P < 0.05.

To verify the molecular mechanisms underlying the anti-senescent “memory” effect of RSV and MET, we knockdown the protein expression of SIRT1 prior to RSV or MET treatment during the incubation in HG for 3 days followed by NG for another 3 days. As is shown in Fig 4, SIRT1 siRNA transfection prior to RSV treatment significantly increased the protein expression of p300, Ac-P53, and p21, as well as decreased SIRT1 and SMP-30 (*P < 0*.*05*; S2 Fig and Fig 4A–4F), while knockdown of SIRT1 prior to MET treatment induced a similar pattern of change in protein expression (*P < 0*.*05*; S2 Fig and 4G–4L). Moreover, Knockdown of SIRT1 not only suppressed the deacetylase activity of SIRT1 (*P < 0*.*05*; Fig 4M and 4N), but also increased the percentage of SA β-Gal staining (*P < 0*.*05*; Fig 4O and 4P) in both RSV- and MET-treated cells.

**Fig 5:**
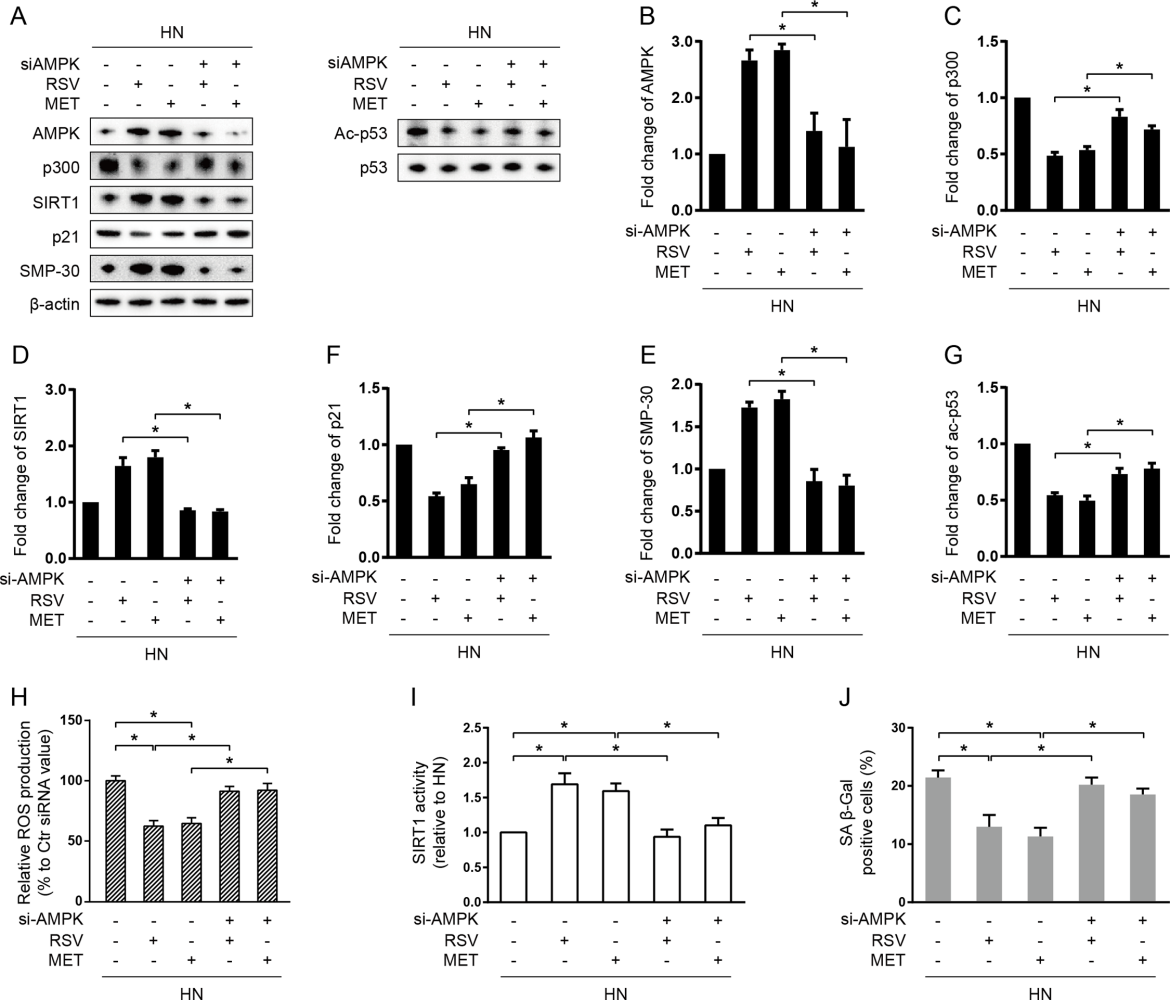
AMPK knock down abolished resveratrol- and metformin-mdiated SIRT1 activation. HUVECs were transfected with AMPK siRNA (si-AMPK) and incubated in HG for 3days and then NG for 3 days. Resveratrol (RSV) or metformin (MET) was added into culture media during the entire 6 days of subculture. Immunoblotting and quantification of p300, SIRT1, p21, Ac-p53, and p53 protein expression (A-G). For p300, SIRT1, and p21, values were normalized to β-actin; for Ac-p53, normalized to total p53. (H) Relative ROS production. (I) Deacetylase activity of SIRT1. (J) SA β-Gal positive positivity. *P < 0.05.

To further investigate whether AMPK play a role in RSV- and MET-induced SIRT1 activation, we transfected AMPK siRNA (si-AMPK) prior to RSV and MET treatment. It was shown that silencing of AMPK expression diminished SIRT1 activation and its downstream signaling (Fig 5A–5G and 5I). Moreover, it abolished the protective effects of RSV and MET against enhanced oxidative stress (Fig 5H) and accelerated cellular aging (Fig 5A, 5E and 5J), suggesting that the effects of RSV and MET on SIRT1 signaling is AMPK-dependent.

**Fig 6:**
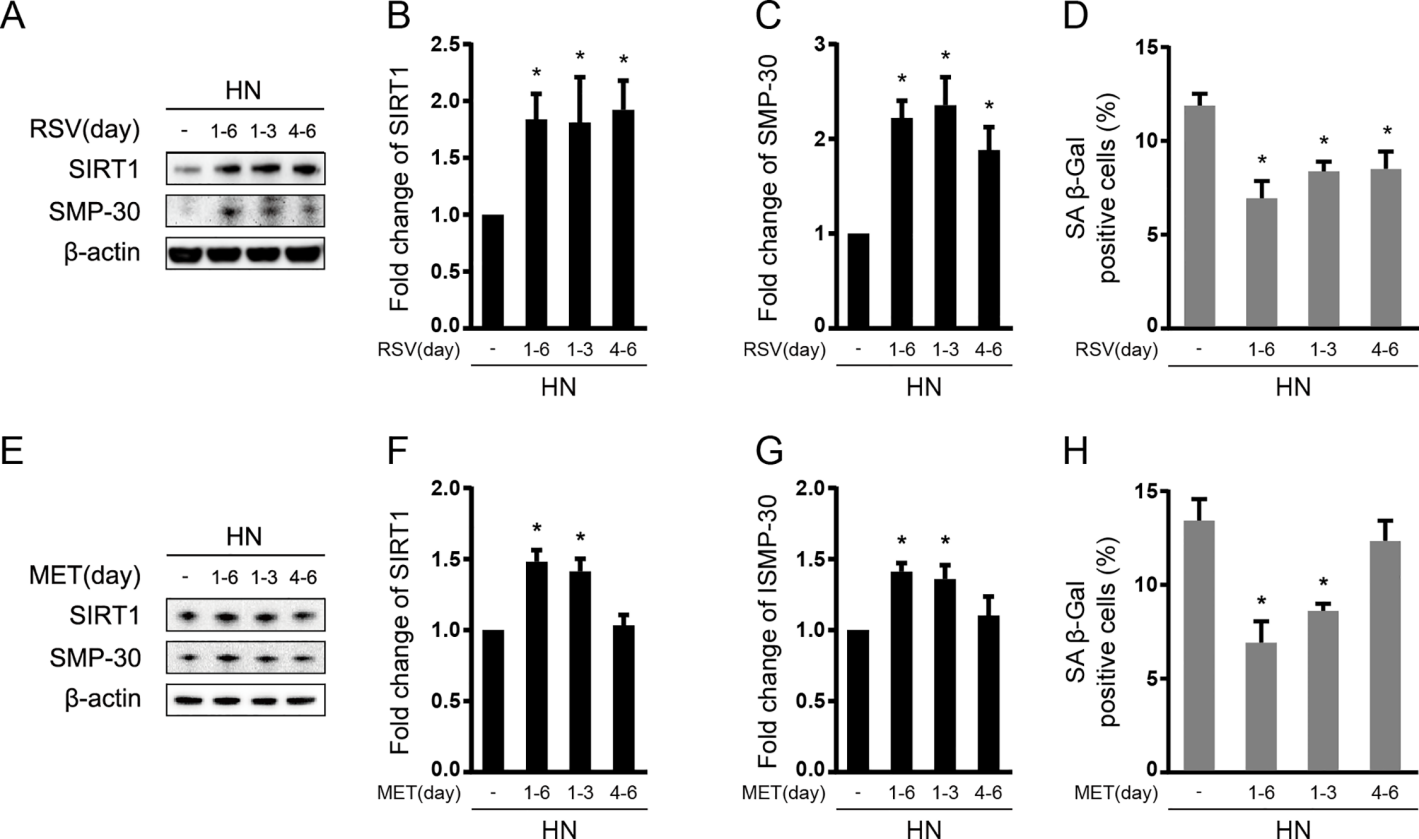
Effect of treatment duration and time points on senescent “memory”. HUVECs in HN group were treated with RSV or MET during the first 3 days of HG incubation (RSV_1-3_ or MET_1-3_), the last 3 days of NG incubation (RSV_4-6_ or MET_4-6_), or during 3 days of HG followed by 3 days of NG incubation (RSV_1-6_ or MET_1-6_). HUVECs in HN group without treatment of RSV or MET were used as control. (A-C) Immunoblotting and quantification of SIRT1 and SMP-30 protein expression in HUVECs treated with or without RSV. Values were normalized to β-actin. (D) The percentage of SA β-Gal positive cells in HUVECs treated with or without RSV. (E-G) Immunoblotting and quantification of SIRT1 and SMP-30 protein expression in HUVECs treated with or without MET. Values were normalized to β-actin. (H) The percentage of SA β-Gal positive cells in HUVECs treated with or without MET. *P < 0.05 compared to HN without RSV or MET treatment.

In the end, to identify whether the protective roles of MET and RSV against transient HG-induced “memory” phenomenon were dependent on the start time and/or duration of treatment, we treated HUVECs in HN group with RSV and MET during the first 3 days of HG incubation (RSV_1-3_ or MET_1-3_), the last 3 days of NG incubation (RSV_4-6_ or MET_4-6_), or during 3 days of HG followed by 3 days of NG incubation (RSV_1-6_ or MET_1-6_). For cells treated with RSV, all the three subgroups (RSV_1-3_, RSV_4-6_, and RSV_1-6_) exhibited significant increases in SIRT1 and SMP-30 protein expression (*P < 0*.*05*; Fig 6A–6C), and a significant decrease in SA β-Gal staining (*P < 0*.*05*; Fig 6D), compared with HN without RSV treatment. For cells treated with MET, the protein levels of SIRT1 and SMP-30 were highly upregulated in MET_1-3_, and MET_1-6_ (*P < 0*.*05*; Fig 6E–6G), while SA β-Gal staining was significantly suppressed in these two groups (*P < 0*.*05*; Fig 6H), in comparison to HN without MET treatment. Notably, the expression of SIRT1 and SMP-30 in MET_4-6_ remained unchanged compared to HN without MET treatment (Fig 6E–6G). Concomitantly, MET_4-6_ failed to alleviate cell senescence (Fig 5H).

## Discussion and Conclusion

### Senescent “memory” was mediated by persistently increased acetylation of p53 at K382

Metabolic memory is a novel concept that, due to prior exposure to hyperglycaemia, diabetic vascular complications may continuously develop despite of current good glycaemic control [18]. The discovery of “metabolic memory” in both T1D and T2D patients strongly suggested that lowering blood glucose to normal level as early as the onset of diabetes may significantly delay the occurrence of diabetic vascular complications. However, it is not always the case in real clinical practice where many patients recognize their diabetic status through early stages of diabetic vascular complications long after many years of existing uncontrolled hyperglycaemia. Therefore, it is crucial to better understand the molecular mechanisms underlying this phenomenon so as to identify novel therapeutic modalities for early intervention.

**Fig 7:**
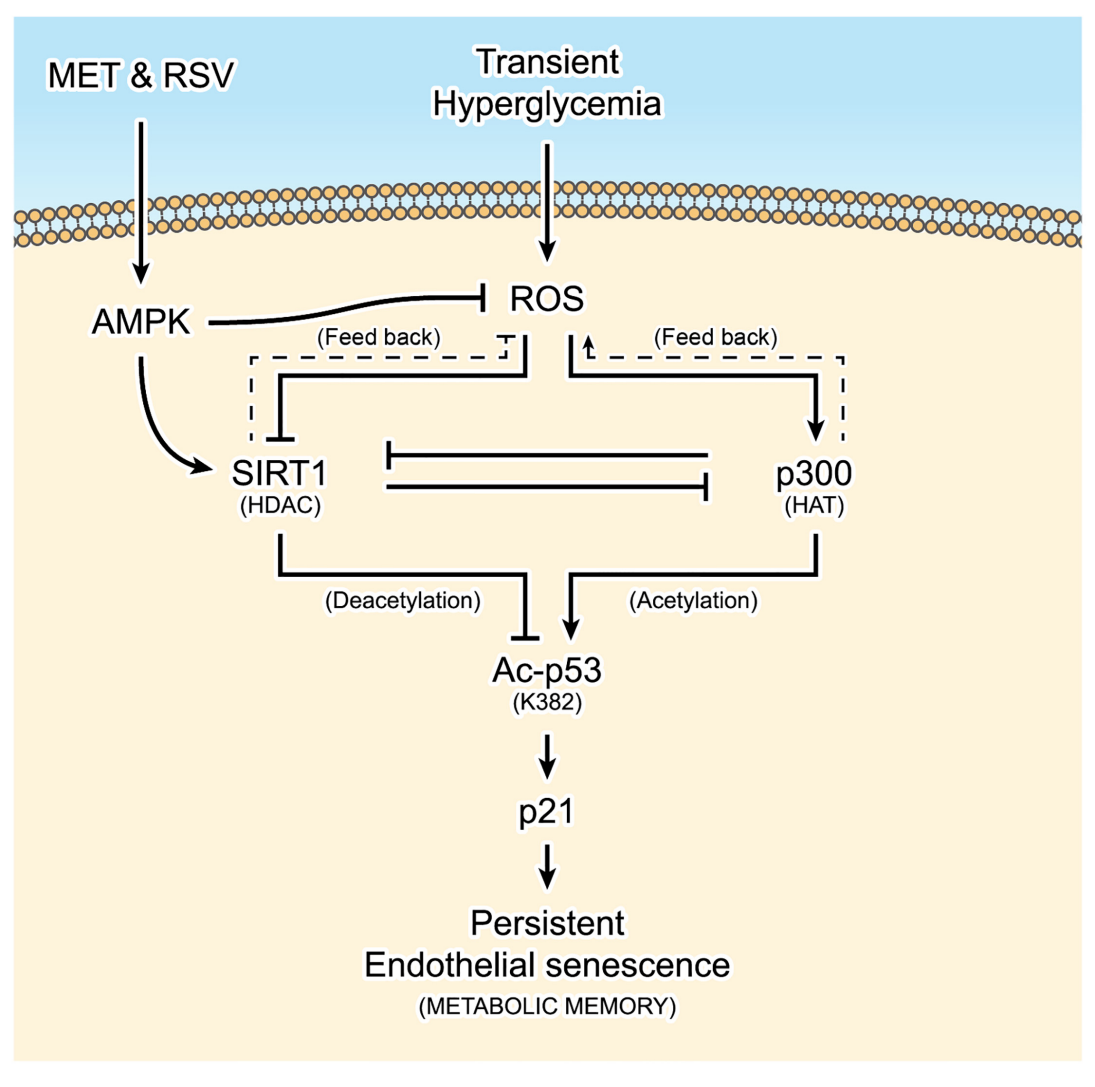
Schematic overview summarizing the molecular mechanisms of resveratrol (RSV) and metformin (MET) against the “metabolic memory” of endothelial senescence in HUVECs. SIRT1 (a histone deacetylase, HDAC) could directly promote the deacetylation of acetylated (Ac)-p53 or indirectly suppress the acetylation of nascent p53 through inhibition of p300 (a histone acetyltransferase, HAT) expression, and thereby modulate Ac-p53/p21-mediated endothelial senescence. Incubating cells in High glucose (HG) for 3 days followed by normal glucose (NG) for 3 days (a cellular model of transient hyperglycaemia) enhanced intracellular ROS production, which in turn inhibited the expression and deacetylase activity of SIRT1 and simultaneously increased the protein level of p300. Accordingly, the transcriptional activity of p53 was increased due to sustained acetylation at lysine 382 (K382), and the expression of p21, a downstream gene of p53, was activated, leading to persistent endothelial senescence (a cellular model of “metabolic memory”). Furthermore, resveratrol (RSV) and metformin (MET) could prevent the transient hyperglycaemia-induced senescent “memory” via AMPK-dependent ROS reduction and SIRT1 activation.

In this study, we used a well-established cellular model of “metabolic memory” by incubating HUVECs in HG for 3 days followed by NG for the remaining 3 days (HN group) [25]. We showed that the level of HUVEC senescence in HN group, represented by the percentage of SA β-Gal positive cells, was significantly higher than that in NG group, and equivalent to those kept in HG media for consecutive 6 days (Fig 1A). This trend of accelerated cellular aging was also confirmed by reduced cell proliferation in HN-treated cells (Fig 1B; HN vs. NG at day 3 and day 6, respectively). In addition, the protein expression and deacetylase activity of SIRT1 were concomitantly decreased (Fig 1D and 1E). Since it has been demonstrated that HG stimulation could induce strong intracellular oxidative stress, which may in turn suppress SIRT1 deacetylase activity on ac-p53 [31], we closely monitored ROS production in all the experimental conditions. As anticipated, HN treatment induced significantly higher level of ROS compared to NG treatment, suggesting a potential link between ROS generation and SIRT1 inhibition in the context of senescent “memory”. Notably, these durable effects of prior HG exposure on ROS generation, SIRT1 expression and activity, as well as cellular aging were not mimicked by D-mannitol, an osmotic control for D-glucose (Fig 1A–1D), indicating that a short-term HG stress induced glucotoxicity and subsequent ROS-mediated SIRT1 inhibition may be primarily responsible for the occurrence of “metabolic memory”. Previously, studies have demonstrated that epigenetic histone post-transcriptional modifications, such as alterations in histone lysine acetylation by HATs and HDACs, are critical to the development of “metabolic memory” [18,32,33]. Nevertheless, there is no report showing that acetylation of non-histone proteins may also exert similar functions. Accordingly, we tested whether the acetylation status of p53 was associated with “metabolic memory” of cellular senescence. Our data showed that 3 days of HG treatment significantly increased the acetylation of p53 at K382 and its downstream target gene p21, which were not diminished by subsequent 3 days of NG incubation. These persistent changes on protein expression in HN-treated cells were comparable to those in HG-treated cells (Fig 1E and 1I). Although, previously, Ac-p53 (K382)-mediated transcriptional activation of p21 has been reported to mediate endothelial senescence in response to high glucose stimulation [14–16], our study demonstrated, for the first time, that transient HG stimulation was strong enough to induce long-lasting acetylation of p53 at K382, and resulted in prolonged cellular senescence even after restoration to normal glucose media, indicating sustained acetylation of non-histone protein as a novel molecular mechanism regulating the occurrence of senescent “memory” (Fig 7).

### Transient HG-induced sustained p53 acetylation was controlled by a delicate balance between SIRT1 and p300

Since p300 and SIRT1 have been shown to be closely associated with the acetylation status of p53 [8–13], we subsequently tested whether Ac-p53-mediated prolonged endothelial senescence was due to functional changes in these up-stream regulators. First of all, we showed that SIRT1 and p300 were inversely regulated in response to transient HG stimulation in HN group (Fig 1F–1H). Then, we analyzed their potential roles on Ac-p53 through gain- and loss-of-function assays. Overexpression of SIRT1 in HN group significantly enhanced the expression and deacetylase activity of SIRT1, and attenuated the Ac-p53-mediated transactivation of p21 (Fig 2A–2F). In the same manner, siRNA knockdown of p300 in HN group also significantly inhibited the acetylation of p53 at K382, resulting in suppression of p21 expression (Fig 2G–2L). In either case, suppression of p53/p21 pathway led to increased SA β-Gal staining (a phenotypic marker of cellular senescence) and reduced the expression of anti-senescence marker SMP-30 (a molecular marker of cellular senescence) in HN-treated HUVECs (Fig 2A, 2E, 2G, 2K, 2O and 2P). Therefore, these data strongly implied that senescent “memory” was mediated by both SIRT1/p53/p21 and p300/p53/p21 pathways.

Interestingly, SIRT1 overexpression inhibited p300 (Fig 2A and 2B), and, in contrast, p300 silencing also enhanced SIRT1 expression (Fig 2G and 2I), implying a potential interaction between SIRT1 and p300 in addition to their respective roles on consensus down-stream target genes. Furthermore, ROS generation was also blunted by both pSIRT1 and si-p300 transfection (Fig 2O and 2R), indicating the existence of feed-back regulations from SIRT1 and p300 towards augmented oxidative stress in transient HG stimulation (Fig 7). Indeed, these results were in accordance to a recent finding in which SIRT1 and p300 exhibited a balancing role on each other and they oppositely regulated oxidative stress pathways in HG-treated human microvascular endothelial cells through acetylation of FOXO1 [34]. Although the authors showed that HG induced endothelial senescence via SIRT1/p300/FOXO1-mediated reduction of mitochondrial antioxidant enzyme, whereas, neither did they address the relationship between endothelial senescence and “metabolic memory”, nor they investigated the therapeutic effects of metformin and resveratrol on inhibiting the “memory” phenomenon of endothelial senescence. Hence, our findings collectively indicated two previously undescribed mechanisms with regard to endothelial senescence: (1) SIRT1 could either directly deacetylate Ac-p53 or indirectly reduce the acetylation of p53 at K382 through suppressing p300 expression, leading to deacetylated inactivation of p53 and subsequent suppression of p21-mediated cellular senescence (SIRT1/p300/p53/p21-mediated endothelial senescence); (2) A short term of HG stress may provoke sustained HUVEC senescence (senescent “memory”) through long-lasting alterations on intracellular ROS generation and SIRT1/p300/p53/p21 signaling.

### Resveratrol and metformin attenuated “metabolic memory” of endothelial senescence via SIRT1-mediated signaling

Resveratrol (RSV) and metformin (MET) are commonly available anti-diabetic drug and dietary supplement, both of which exert endothelial protective effects independent of their glucose-lowing mechanisms. In particular, they are shown to activate SIRT1 mediated pathways, so as to promote cell growth, proliferation and survival [17,19–24]. Thus, we speculated that MET and RSV may function as potent activators of SIRT1 to prevent senescent “memory”. To test this hypothesis, we supplemented HUVEC media with RSV or MET during 3 days of HG and another 3 days of NG incubation. Not to our surprise, RSV or MET treatment significantly increased the expression and deacetylase activity of SIRT1, accompanied by significant inhibition on Ac-p53/p21-mediated cellular senescence (Fig 3). To further confirm whether these anti-senescent actions of RSV and MET were SIRT1 dependent, we transfected SIRT1 siRNA and then added RSV or MET into NH-treated HUVECs. It was shown that, either in RSV-treated or MET-treated cells, knockdown of SIRT1 significantly decreased the expression and activity of SIRT1, and increased its downstream target genes, including p300, Ac-p53, and p21, compared with those transfected with scramble siRNA (Fig 4A–4N). In addition, SIRT1 siRNA transfection markedly reduced the markers of cellular senescence in HN-treated cells (Fig 4A, 4E, 4G, 4K, 4O and 4P). Furthermore, RSV- or MET-mediated activation of SIRT1 (Fig 3E and 3F) and siRNA-mediated inhibition of SIRT1 (Fig 4A, 4B, 4G and 4H) triggered opposite changes between SIRT1 and p300 protein expression. Collectively, these data suggested that RSV and MET may exert hypoglycemic-independent protective roles against the occurrence of senescent “memory” through SIRT1/p300/p53/p21 pathway.

Previously, Zheng et al. [35] have demonstrated that MET could attenuate HG-induced cellular “memory” of oxidative stress in primary bovine retinal capillary endothelial cells via SIRT1/LKB1/AMPK/ROS pathway. This was the first report showing the involvement of SIRT1, as well as the therapeutic effect of MET, during the occurrence of “metabolic memory”. Similarly, in our study, we observed that RSV and MET treatment suppressed ROS generation through SIRT1 activation in the context of vascular metabolic memory (Fig 5H). Moreover, we showed that AMPK silencing diminished RSV- and MET-induced SIRT1 activation (Fig 5A–5G and 5I) and thus abolished their protective effects against ROS generation and cellular senescence (Fig 5A, 5G and 5J), suggesting that both RSV and MET could protect against senescent “memory” through AMPK-dependent SIRT1 activation and ROS reduction (AMPK/SIRT1/ROS pathway).

Considering that, in previous sections, we had shown that early and continuous RSV or MET treatment (during the entire 6 days of sequential HG and NG incubation) blocked the occurrence of senescent “memory”, then we started to wonder whether treating RSV or MET for shorter periods would convey similar protective effects. Accordingly, HUVECs in HN group were treated with RSV or MET during the first 3 days of HG (RSV_1-3_ or MET_1-3_), the last 3 days of NG (RSV_4-6_ or MET_4-6_), or the whole 6 days of sequential HG and NG incubation (RSV_1-6_ or MET_1-6_). In RSV-treated HUVECs, significant changes in SIRT1 expression and markers of endothelial senescence were observed between untreated and differently treated subgroups, regardless of the start time and duration of treatment (Fig 6A–6D), indicating that RVS could exert both preventive (during 3 days of HG period) and/or therapeutic (during 3 days of NG period) effects through activating SIRT1-mediated signaling. Interestingly, although similar levels of SIRT1 expression and cellular senescence were observed among MET_1-3_ and MET_1-6_ (vs. HN, *P < 0*.*05*; Fig 6E–6H), the SIRT1 expression and cellular senescence in MET_4-6_ remained unchanged compared to untreated HN (Fig 6E–6H). These results may indicate that, while different RSV start time and duration exert comparable protective actions against the transient HG-induced senescent “memory”, early and continuous MET treatment is particularly important for preventing the occurrence of senescent “memory”.

Although in this study, we showed a central role of SIRT1 in modulating hyperglycemic memory of endothelial senescence, it should be carefully noted that the above mentioned conclusions were extracted from an established *in vitro* model of “metabolic memory”, and 3 days of HG followed by 3 days of NG is a relatively short term of observation. Indeed, in real clinical situation, it usually takes years for an individual to develop diabetic vascular complications, and many risk factors and mechanisms are involved during the pathogenesis. Therefore, continuous studies with longer observation period and in multiple biological systems are warranted to further validate these mechanisms, so as to expand its significance and applications. Nonetheless, our findings underscored the concept of “metabolic memory” that changes on some endothelial gene expression cannot be immediately reversed even after normal glucose level were reinstituted. In addition, we proposed a novel perspective that short-term of high glucose insult not only causes long-lasting post-translational modifications on histone proteins (such as H3K9me3 at the promoter of NF-κB [36]), but also induces alterations on non-histone proteins (such as ac-p53 in our study).

Taken together, in current study, we demonstrated the existence of “metabolic memory” of endothelial senescence (senescent “memory”) by showing that transient hyperglycaemic stress provoked persistent premature cellular senescence in macrovascular HUVECs, despite subsequent restoration to normoglycaemia. In addition, we showed that sustained changes on a novel SIRT1/p300/p53/p21 signaling were responsible for the formation of the senescent “memory”. Moreover, metformin and resveratrol prevented the occurrence of “metabolic memory” through the above mentioned SIRT1 axis. Therefore, our study collectively indicated that resveratrol and metformin may serve as promising therapeutic drugs against high glucose-induced “metabolic memory” of endothelial senescence through modulating SIRT1/p300/p53/p21 pathway, so as to further prevent the continuous progression of diabetic vascular complications (Fig 7).

## Supporting Information

S1 Fig The time-course effects of SIRT1 overexpression and p300 knockdown. Cells were transfected with SIRT1 plasmid (pSIRT1) or p300 siRNA (si-p300) and then cultured in HG media for 1 day or 3 days. Immunoblotting and quantification of p300, SIRT1, p21, Ac-p53, and p53 protein expression after pSIRT1 transfection for 1 day (A) or 3 days (B), or after si-p300 transfection for 1 day (C) or 3 days (D). For p300, SIRT1, and p21, values were normalized to β-actin; for Ac-p53, normalized to total p53. *P < 0.05.
(TIF)

S2 Fig The time-course effects of SIRT1 knockdown. Cells were transfected with SIRT1 plasmid (pSIRT1) or p300 siRNA (si-p300) and then cultured in HG media supplemented with RSV or MET for 1 day or 3 days. Immunoblotting and quantification of p300, SIRT1, p21, Ac-p53, and p53 protein expression after RSV treatment for 1 day (A) or 3 days (B), or after MET for 1 day (C) or 3 days (D). For p300, SIRT1, and p21, values were normalized to β-actin; for Ac-p53, normalized to total p53. *P < 0.05.
(TIF)
