# Supplementary figures and images for: Metformin and Resveratrol Inhibited High Glucose-Induced Metabolic Memory of Endothelial Senescence through SIRT1/p300/p53/p21 Pathway
Source: PLoS One. 2015 Dec 2;10(12):e0143814. doi: 10.1371/journal.pone.0143814 (PMC4668014; doi:10.1371/journal.pone.0143814)

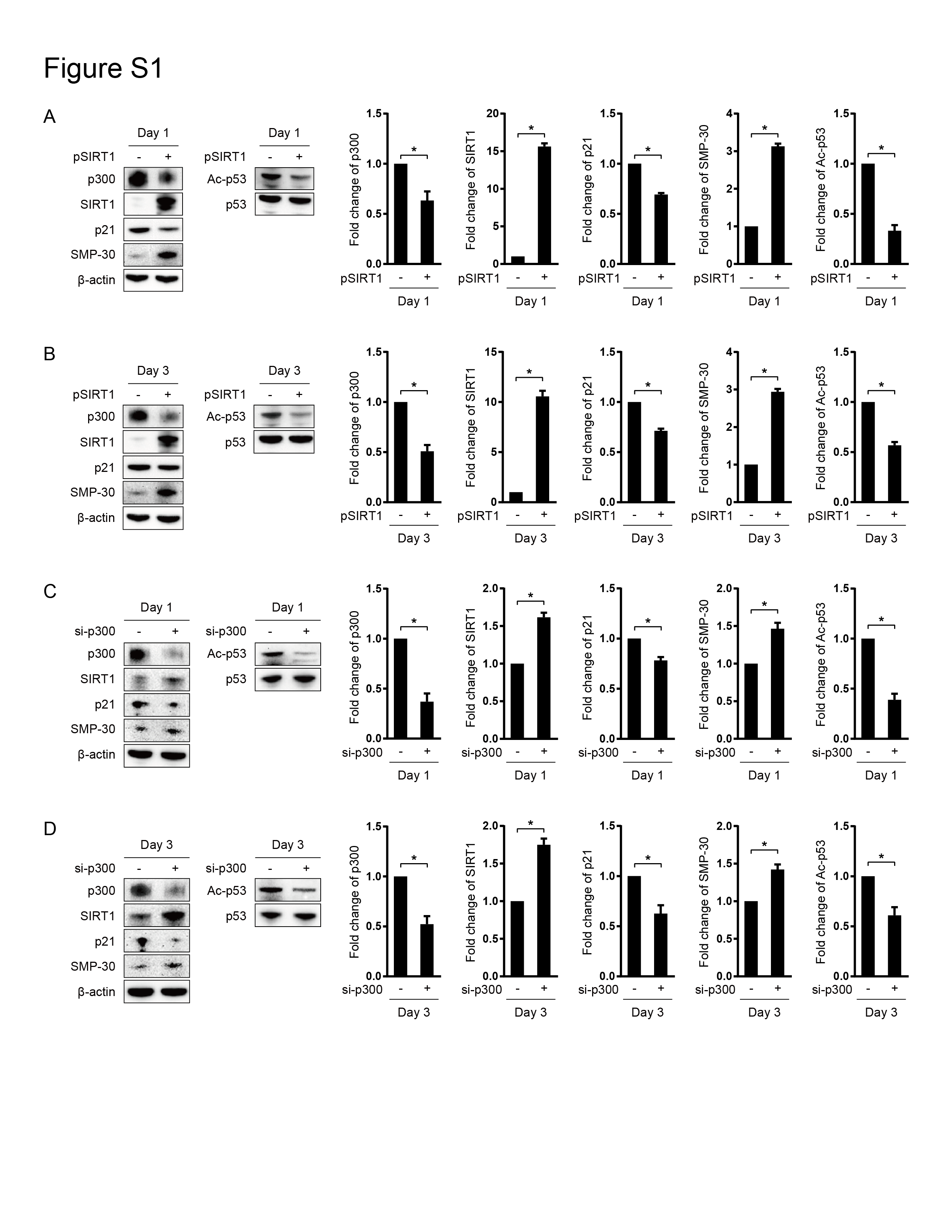

Supplement: S1 Fig — Cells were transfected with SIRT1 plasmid (pSIRT1) or p300 siRNA (si-p300) and then cultured in HG media for 1 day or 3 days. Immunoblotting and quantification of p300, SIRT1, p21, Ac-p53, and p53 protein expression after pSIRT1 transfection for 1 day (A) or 3 days (B), or after si-p300 transfection for 1 day (C) or 3 days (D). For p300, SIRT1, and p21, values were normalized to β-actin; for Ac-p53, normalized to total p53. *P < 0.05. (TIF) [file pone.0143814.s001.tif]

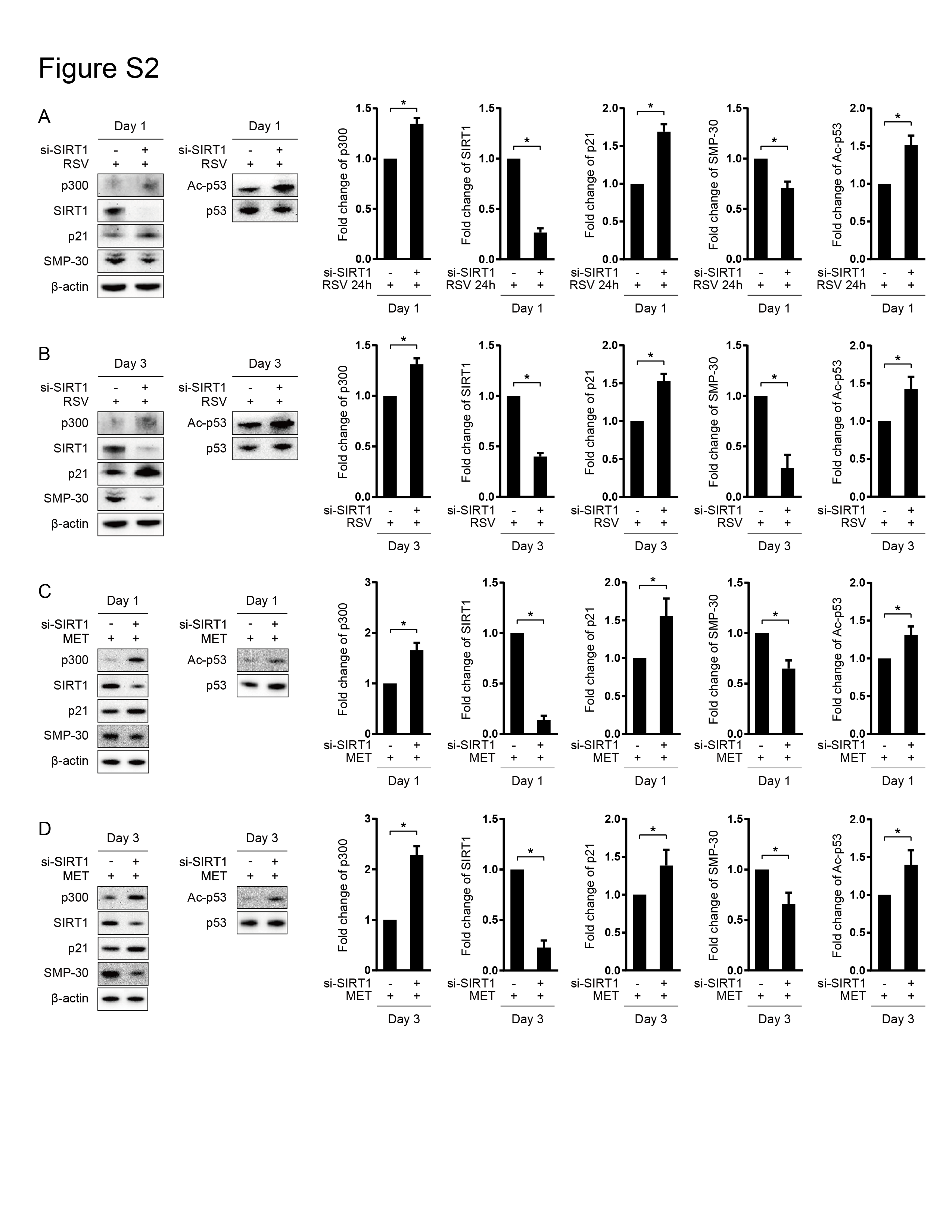

Supplement: S2 Fig — Cells were transfected with SIRT1 plasmid (pSIRT1) or p300 siRNA (si-p300) and then cultured in HG media supplemented with RSV or MET for 1 day or 3 days. Immunoblotting and quantification of p300, SIRT1, p21, Ac-p53, and p53 protein expression after RSV treatment for 1 day (A) or 3 days (B), or after MET for 1 day (C) or 3 days (D). For p300, SIRT1, and p21, values were normalized to β-actin; for Ac-p53, normalized to total p53. *P < 0.05. (TIF) [file pone.0143814.s002.tif]
